# Somatic Symptoms of Depression Lose Association with Mortality upon Adjustment for Frailty: Analysis from the Fitness Haemodialysis Cohort

**DOI:** 10.1155/2023/4518843

**Published:** 2023-06-21

**Authors:** Benjamin M. Anderson, Muhammad Qasim, Gonzalo Correa, Felicity Evison, Suzy Gallier, Charles J. Ferro, Thomas A. Jackson, Adnan Sharif

**Affiliations:** ^1^Department of Nephrology and Transplantation, Queen Elizabeth Hospital, Birmingham, UK; ^2^Institute of Inflammation and Ageing, University of Birmingham, Birmingham, UK; ^3^Institute of Immunology and Immunotherapy, University of Birmingham, Birmingham, UK; ^4^Department of Nephrology, Hospital del Salvador, Santiago, Chile; ^5^Department of Health Informatics, Queen Elizabeth Hospital, Birmingham, UK; ^6^PIONEER HDR-UK Hub in Acute Care, Edgbaston, Birmingham, UK; ^7^Institute of Cardiovascular Sciences, University of Birmingham, Birmingham, UK; ^8^Department of Healthcare for Older People, Queen Elizabeth Hospital, Birmingham, UK

## Abstract

**Introduction:**

The somatic symptom component of depression is associated with increased hospitalisation and mortality and poorer health-related quality of life (HRQOL). However, the relationship of subsets of depression symptoms with frailty and outcomes is not known. This study aimed to (1) explore the relationship between the Clinical Frailty Scale (CFS) and components of depression and (2) their association with mortality, hospitalisation, and HRQOL in haemodialysis recipients.

**Methods:**

We conducted a prospective cohort study of prevalent haemodialysis recipients, with deep bio-clinical phenotyping including CFS and PHQ-9 somatic (fatigue, poor appetite, and poor sleep) and cognitive component scores. EuroQol EQ-5D summary index assessed HRQOL at the baseline. Electronic linkage to English national administration datasets ensured robust follow-up data for hospitalisation and mortality events. *Findings*. Somatic (*β* = 0.067; 95% C.I. 0.029 to 0.104; *P* < 0.001) and cognitive (*β* = 0.062; 95% C.I. 0.034 to 0.089; *P*<0.001) components were associated with increased CFS scores. Both somatic (*β* = −0.062; 95% C.I. −0.104 to −0.021; *P*<0.001) and cognitive (*β* = 0.052; 95% C.I. −0.081 to −0.024; *P* < 0.001) scores were associated with lower HRQOL. Somatic scores lost mortality association on addition of CFS to the multivariable model (HR1.06; 95% C.I. 0.977 to 1.14; *P*=0.173). Cognitive symptoms were not associated with mortality. Neither the component score was associated with hospitalisation on multivariable analyses.

**Conclusions:**

Both somatic and cognitive depression symptoms are associated with frailty and poorer HRQOL in haemodialysis recipients but were not associated with mortality or hospitalisation when adjusted for frailty. The risk profile of depression somatic scores may be related to overlap with symptoms of frailty.

## 1. Introduction

Frailty is a syndrome of increased vulnerability to poor resolution of homeostasis after a stressor event [1]. It is associated with poor patient outcomes including mortality, hospitalisation, and disability [2]. It is commonly defined using the Clinical Frailty Scale (CFS) [3], a simple global measure of frailty based upon activities of daily living after clinical interview. In prevalent haemodialysis cohorts, estimates of frailty prevalence range from 26 to 54% using the CFS [4, 5] and it is associated with mortality and hospitalisation [4].

Depression is under-recognised in haemodialysis populations [6] and is associated with increased mortality and hospitalisation [7–12]. The Patient Health Questionnaire-9 (PHQ-9) has been validated in dialysis recipients with 92% sensitivity and 92% specificity for depression [13]. However, our previous work has not been able to demonstrate links between PHQ-9 depression and these outcomes (submitted for peer-review). Work elsewhere has explored dividing depression scores into somatic and cognitive symptom components [14]. The somatic symptom subset of the Beck Depression Inventory (BDI) has been associated with mortality and hospitalisation in haemodialysis recipients in Dutch [15] and Jordanian [16] haemodialysis recipients. In the Dutch cohort, depression, somatic symptoms, and cognitive symptoms were all associated with lower quality of life [15].

Frailty has been associated with poorer quality of life in Brazilian [17] and UK [18] nondialysis CKD cohorts. McAdams-DeMarco and colleagues found that self-rated fair/poor quality of life was more likely in frail haemodialysis recipients and that frailty was associated with worsening quality of life over time [19]. A decline in quality of life in frail haemodialysis recipients was also observed in a small Canadian cohort [20].

Whilst there are reports of a reciprocal relationship between frailty and depression [21, 22], no such exploration of the relationship between frailty versus the somatic and cognitive components of depression has taken place. This may be important as the somatic symptoms of depression such as tiredness, poor sleep, and lack of appetite show considerable overlap with those of frailty. Therefore, the aims of this study were to (1) explore the relationship between frailty and components of depression and (2) assess the association between the components of depression with mortality, hospitalisation, and quality of life in haemodialysis recipients.

## 2. Materials and Methods

### 2.1. Study Design

Frailty Intervention Trial iN End-Stage patients on haemodialysiS (FITNESS) is a cohort multiple randomised controlled trial (cmRCT) [23] split into two stages, for which the full protocol has been published elsewhere [24]. The study protocol was subject to favourable opinion by the South Birmingham Research Ethics Committee (Ref: 17/WM/0381) and institutional review board assessment of University Hospitals Birmingham NHS Foundation Trust (RRK6082). Here, we report data from the first stage of the FITNESS project, a cohort study with extensive baseline phenotyping for frailty and other bio-clinical parameters. The study is reported in accordance with STROBE guidelines [25].

### 2.2. Study Setting

This study was performed in a single nephrology centre in Birmingham, England, which oversees one in-hospital dialysis unit and ten private-provider satellite units across urban and rural settings across the West Midlands, with consequent diversity of ethnic and socioeconomic groups. Patient eligibility was ascertained using hospital electronic patient records (EPR) and liaison with healthcare professionals at each dialysis unit. Eligible patients were contacted in person on dialysis. Study investigators provided written and verbal information to prospective participants and answered any queries. Sufficient time was allowed to consider the information, before willing patients gave written informed consent to participate.

### 2.3. Eligibility Criteria

Inclusion criteria included adults aged 18 and over, anyone receiving regular haemodialysis for at least 3 months' duration, and the ability to give informed consent. Patients were excluded if they received inpatient care within 4 weeks of recruitment unless for the purposes of vascular access, to avoid potential confounding by frailty associated with hospitalisation.

### 2.4. Baseline Assessment

All study participants underwent baseline assessment during one of their usual scheduled dialysis sessions. Participants were not assessed on the first dialysis session after the weekend interval (i.e., Monday or Tuesday), to prevent confounding by the longer interdialytic interval before assessment. Where participants dialysed twice weekly, the dialysis session after the shortest interval was chosen for baseline assessment.

Study investigations are described in detail in our methodology study [24]. Briefly, prior to connection to dialysis, participants were invited to complete a timed 4 metre walk from standing and to test bilateral hand-grip strength via a dynamometer (Takei Grip D, Takei Scientific Instruments Co. Ltd., Japan). Montreal cognitive assessments (MoCAs [26]) were also performed prior to dialysis connection. When connected to dialysis, patients were clinically interviewed, collecting demographic, social and medical history data, alongside assessment of activities of daily living (ADL) disability, and frailty-specific questionnaires. Depression symptoms were assessed using the PHQ-9 questionnaire [13]. The Physical Activity Index was derived from the GP physical activity (GPPAQ) [27] questionnaires via a validated formula to give a global measure of participant physical activity. [27, 28] Electronic patient records were interrogated for data upon biochemistry, dialysis adequacy, comorbidities, prescribed medications, alongside dialysis vintage, and previous renal replacement therapies. Self-reported change in health (henceforth “health change”) was assessed with the question “How has your health changed in the last year?” with potential responses of “Better,” “The Same,” or “Worse” [29]. The English Index of Multiple Deprivation 2015 (IMD) was used to assess socioeconomic deprivation [30]. This is a national multiple deprivation model calculated from multiple socioeconomic data points at a postal code (Zip code) level. A composite score is obtained and split into quintiles of deprivation, with 1 representing the most deprived and 5 the least deprived area, respectively.

PHQ-9 somatic component scores included questions 3 (“Trouble falling or staying asleep or sleeping too much”), 4 (“Feeling tired or having little energy”), and 5 (“Poor appetite or overeating”). Cognitive component scores included questions 1 (“Little interest or pleasure in doing things”), 2 (“Feeling down, depressed, or hopeless”), 6 (“Feeling bad about yourself or that you are a failure or have let yourself or your family down”), 7 (“Trouble concentrating on things, such as reading the newspaper or watching television”), 8 (“Moving or speaking so slowly that other people could have noticed? Or the opposite-being so fidgety or restless that you have been moving around a lot more than usual”), and 9 (“Thoughts that you would be better off dead or of hurting yourself in some way”) [14].

The Clinical Frailty Scale was obtained by interpretation of ADL questionnaire responses, with possible responses of 1–9. A CFS of 1 represented very fit and 8 severely frail. A CFS score of 9 was attributed to those who were terminally ill but not overtly frail. A CFS score of ≥5 was considered frail.

### 2.5. Outcomes

Mortality and cause of death data were obtained by electronic record linkage to the Office of National Statistics (ONS), a UK-wide database of death certification. Hospitalisation data were obtained via Hospital Episode Statistics (HES), a clinical coding database containing all secondary care episodes in any English NHS hospital. Hospital admissions were defined as any hospital episode lasting ≥1 night. Transfers between hospitals were treated as one continuous admission, and length of stay in such episodes was calculated from admission at the initial hospital to discharge from the final hospital.

Health-related quality of life was assessed using Euroqol EQ-5D-3L. To allow global assessment of HRQOL, EQ-5D-3L responses were converted into a single-measure EQ-5D summary index via a standardised formula, validated in UK populations [31]. The EQ-5D summary index score ranges between 1 (if no HRQOL deficits reported) and −0.716 (if extreme deficit in every domain).

### 2.6. Recruitment

A power calculation was based upon US data demonstrating an association of frailty with an adjusted risk ratio of 2.24 for 1-year mortality and 1.56 for 1-year mortality/hospitalisation in haemodialysis recipients [32]. We assumed a nonfrail risk of mortality and mortality/hospitalisation to be 5% and 40%, respectively. Powered to 0.8 and with a confidence interval of 0.95, a sample size of 602 was therefore calculated for 1-year mortality and 150 patients for 1-year mortality/hospitalisation. Upon discussion and agreement with the sponsor, however, recruiting 602 participants was not felt to be feasible in this single centre. As such a revised target of 500 participants was set with follow-up beyond 1 year.

### 2.7. Statistics

Statistical analysis was performed using STATA 17 (StataCorp 2019, Stata Statistical Software: Release 17, College Station, TX: StataCorp LLC) and R version 4.0.4 (R Foundation for Statistical Computing, Vienna, Austria). Categorical data were presented as numbers and percentages, and continuous variables were reported as medians and interquartile ranges (IQRs).

Time-to-event outcomes were analysed with Cox's proportional hazards model. The proportional hazard assumption was checked via interrogation of the log-negative-log plots of the within-group survivorship functions versus log time. Furthermore, we compared Kaplan–Meier (observed) with Cox (expected) survival curves for study variables (reported as hazard ratios (HRs) with 95% confidence intervals (CIs)).

Linear regression analyses explored the relationship between continuous frailty and PHQ-9 scores and between these same scores and EQ-5D summary index. The linearity assumption was checked by visually comparing plots of observed values by linear and LOWESS fit and by plotting observed versus predicted residual values. Linear regressions were performed unadjusted and as a series of adjusted models based upon *a priori* covariables selected for known or suspected relationship with the outcome of interest. Due to the number of covariables, adjusted models were constructed in a predetermined stepwise manner. For frailty, model 1 included depression, age, gender, and ethnicity. Model 2 added education level, social support, and IMD quintile. Model 3 added Charlson Index (CKD excluded), MoCA score, smoking status, self-rated change in health, and overall health. Model 4 added use of walking aids, Physical Activity Index, slow timed walk, and low grip strength.

Regressions for the EQ-5D summary index were performed unadjusted and subject to a separate set of *a priori* covariable models, based upon known or suspected relationship with HRQOL, and covariables found to significantly associate with frailty and/or PHQ-9 scores were also added to reduce confounding. Due to the large number of covariables identified, models were constructed in a predetermined stepwise approach. Model 1 included age, gender, ethnicity, education level, social support, IMD quintile, and employment status. Model 2 added to these haemodialysis vintage, Charlson comorbidity index (CKD omitted), haemoglobin, Kt/V, and current use of antidepressant medication. Model 3 added use of walking aids, slow walking (or inability to walk), and Physical Activity Index. Model 4 added EQ self-rated health today (continuous score from 0 to 100) and self-rated health change.

Count data were explored by negative binomial regression, death-censored and offset by length of follow-up, to give incidence rate ratios (IRRs). Negative binomial distribution was confirmed by interrogation of means and variances and visual inspection of observed versus expected distribution plots. Zero-truncated negative binomial regressions were performed for nights per admission, as by definition these could not equal zero.

We performed both unadjusted and adjusted negative binomial and Cox regressions. Covariables for adjusted analyses were selected *a priori* based upon a proven or suspected relationship with hospitalisation and/or mortality (age, sex, ethnicity (grouped into white, south Asian, black, and other ethnicities), body mass index, index of multiple deprivation, Charlson comorbidity index (CKD omitted), number of hospitalisation episodes, number of medications, smoking status, serum albumin, use of walking aids, dialysis vintage, self-reported change in health, and kidney transplant wait-listing). Furthermore, adjusted models were performed using the aforementioned covariables plus the addition of the CFS score. No transplant-listed participants died within 1 year of recruitment, so this covariable was omitted in the final logistic regression models for 1-year mortality.

A dummy variable was used to handle missing IMD quintile data. All other covariables were assumed missing at random as they had <1% data missing and were therefore handled via listwise deletion. Statistical significance was set at a *P* value <0.05.

## 3. Results

### 3.1. Study Cohort Demographics

**Figure 1:**
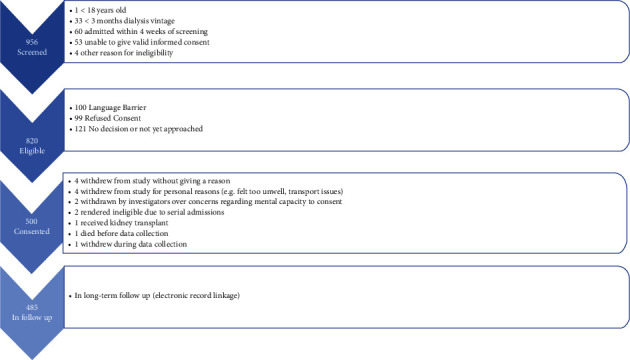
PRISMA flow diagram of study recruitment.

Figure 1 shows the PRISMA study flow of participant recruitment to the FITNESS study, with 485 prevalent haemodialysis patients with baseline frailty assessments and data linkage. Follow-up was 678 days (interquartile range: 531–812 days), with minimum potential follow-up of 365 days from recruitment. Baseline demographics are described in detail elsewhere [5].  Table 1 shows key demographics stratified by frailty status at study recruitment.

### 3.2. PHQ-9 Somatic and Affective Scores

The median PHQ-9 somatic and cognitive scores were 3 (IQR 1, 6) and 1 (IQR 0, 5), respectively. Scores ranged from 0 to 9 for the PHQ-9 somatic score and 0–18 for the PHQ-9 cognitive score.

### 3.3. Relationship with Frailty

**Figure 2:**
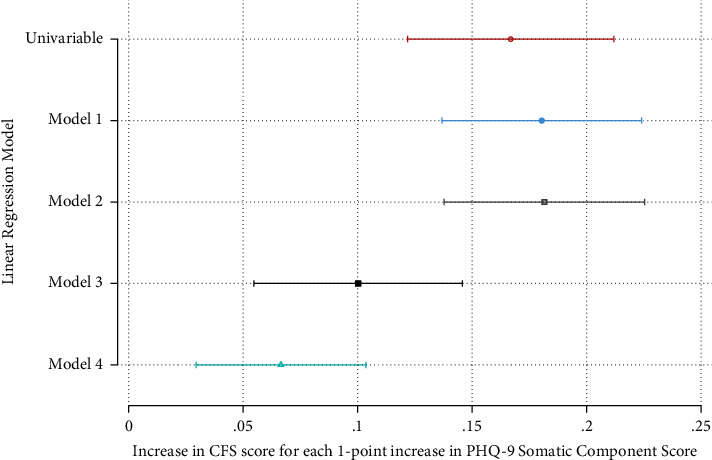
Association of the PHQ-9 somatic component score with the CFS score on simple and multiple linear regression analyses. Model 1 includes depression, age, gender, and ethnicity. Model 2 adds the education level, social support, and IMD quintile. Model 3 adds the Charlson Index (CKD excluded), MoCA score, smoking status, self-rated change in health, and overall health. Model 4 adds use of walking aids, physical activity index, slow timed walk, and low grip strength.

**Figure 3:**
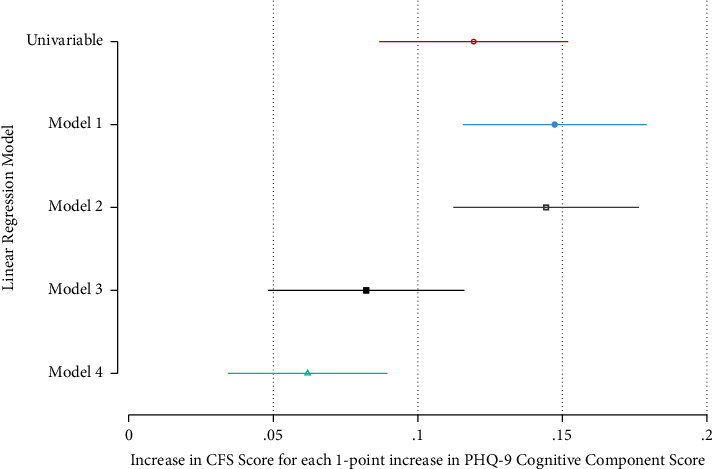
Association of the PHQ-9 cognitive component score with the CFS score on simple and multiple linear regression analyses. Model 1 includes depression, age, gender, and ethnicity. Model 2 adds the education level, social support, and IMD quintile. Model 3 adds the Charlson Index (CKD excluded), MoCA score, smoking status, self-rated change in health, and overall health. Model 4 adds use of walking aids, physical activity index, slow timed walk, and low grip strength.

Figures 2 and 3 show that both somatic and cognitive scores were positively associated with the CFS score on simple and all multiple linear regression models. Full results of the final models for somatic and cognitive scores are shown in Supplementary Tables 1 and 2.

### 3.4. Association with Mortality

**Figure 4:**
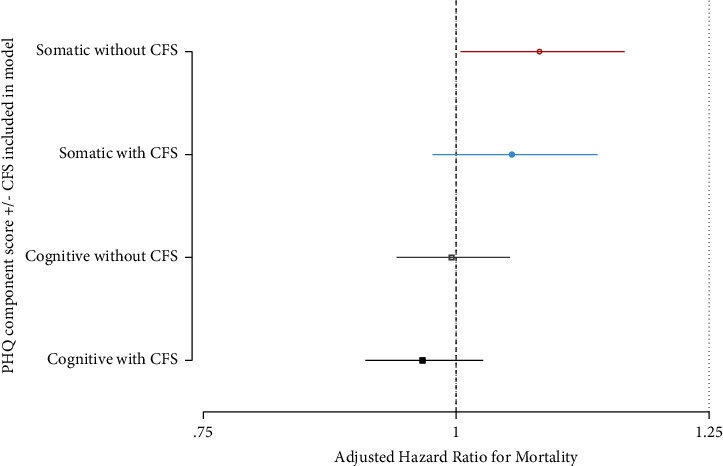
Adjusted hazard ratios of PHQ-9 somatic and cognitive component scores' association with mortality after Cox regression, both with and without inclusion of CFS.

PHQ-9 somatic component was associated with mortality on univariable analysis (HR 1.10; 95% C.I. 1.02, 1.17; *P*=0.007), but cognitive component was not (HR 1.00; 95% C.I. 0.950, 1.05; *P*=0.977).  Figure 4 shows that the somatic component retained this association on multivariable analysis with CFS omitted but lost the significance on addition of CFS to the model. The affective component was not associated with mortality regardless of CFS inclusion. Full adjusted models are shown in Supplementary Tables 3–6.

### 3.5. Association with Hospital Admissions

The PHQ-9 somatic score was associated with increased rates of hospital admissions on univariable analysis (IRR 1.05; 95% C.I. 1.01, 1.10; *P*=0.015) but not upon multivariable analysis (IRR 1.00; 95% C.I. 0.96, 1.04; *P*=0.993).

PHQ-9 cognitive scores were not associated with rates of hospital admissions on either univariable (IRR 1.02; 95% C.I. 1.00, 1.06; *P*=0.103) or multivariable analyses (IRR 0.98; 95% C.I. 0.95, 1.01; *P*=0.194). Fully adjusted model results for somatic and cognitive scores are shown in Tables 2 and 3, respectively; of the *a priori* covariables in these models, age, CFS, the Charlson Index, number of previous admissions, and walking aid use were all associated with higher rates of admissions, while black ethnicity was associated with lower admission rates when included in both cognitive and somatic component score models. Supplementary Tables 7 and 8 show that omission of the CFS score from multivariable models did not significantly alter results.

### 3.6. Association with Quality of Life

Increases in both somatic and cognitive components of PHQ-9 were associated with lower EQ Summary Index scores on fractional regression, as shown in Table 4. Furthermore, increasing CFS scores were also associated with significant reductions in EQ Summary Index on all models, independent of somatic and cognitive component scores. Fully adjusted fractional regression model results are shown in Supplementary Tables 9 and 10.

## 4. Discussion

Both depression and frailty have been associated with mortality and hospitalisation in dialysis patients [7–12]. However, previous work in the FITNESS cohort has shown that depression was not significantly associated with either of these important negative outcomes [33]. The somatic and cognitive components of depression have also been associated with hospitalisation and mortality [15, 16], though their relationship with frailty has not been explored. Here, we show that both the somatic and cognitive components of depression are associated with increasing frailty. Only somatic symptoms were associated with increased mortality and hospitalisation in univariable analyses but lost these associations on multivariable analyses. Both cognitive and somatic symptoms were associated with lower health-related quality of life. Lack of association between somatic depression symptoms and hospitalisation or mortality contradicts reports elsewhere, but the association with poorer quality of life indicates that these symptoms remain important to haemodialysis recipients.

Work within the FITNESS cohort has shown that while PHQ-9 scores associate with frailty, they predict neither admissions nor mortality in prevalent haemodialysis recipients [33]. Schouten and colleagues showed a differential risk profile between the somatic and cognitive components of the BDI [15]. Somatic, but not cognitive, symptoms were associated with all-cause mortality, whereas both dimensions were associated with hospitalisation and poorer HRQOL [15]. Khalil et al. found a significant association between both somatic and cognitive component scores of the BDI with mortality and hospitalisation in a Jordanian haemodialysis cohort [16]. In the FITNESS cohort, however, the cognitive component did not associate with hospitalisation or mortality. Furthermore, while the somatic component did associate with mortality on multivariable analysis, it lost this association on addition of frailty into the model. We must exercise caution when comparing data across national and socio-cultural boundaries, obtained using different methodologies and heterogenous depression scores. However, neither the Dutch nor Jordanian cohorts included a frailty measure in their analyses [15, 16]. Given the overlap between symptoms of frailty and somatic depression symptoms, we may speculate that the somatic depressive symptoms may represent a surrogate marker for frailty in haemodialysis recipients. This may explain the loss of mortality association upon the addition of a *de facto* measure of frailty and a more powerful associate with negative outcomes. These novel findings indicate some of the complexity inherent to frailty assessment in heavily comorbid populations such as haemodialysis.

To our knowledge, FITNESS is amongst the first studies to explore relationships between frailty and depression symptom subsets in a large prospective haemodialysis cohort. Strengths of the study include the large cohort size, prospectively recruited, with diversity of population representative of our local populace [34]. The cohort is deeply phenotyped, allowing for a broad range of medical, social, and lifestyle factors to be included in our analyses. Furthermore, electronic data linkage ensures robust data capture of hospitalisation and mortality. However, we must advise caution in applying our data to non-English populations; validation of our findings elsewhere is required. We have adjusted for many potential confounders in our analyses, but we must be cautious about overfitting the models to our cohort. Covariables were added to our models in a stepwise manner to mitigate for this. As complexity of the models increased, the effect of the independent variable was attenuated, but we would argue that the inferences remained the same regardless of the model used. Limitations also include the single baseline data collection for frailty and depression phenotyping, both frailty and depression are dynamic states, and serial measurements would arguably improve both accuracy and analytical detail [10, 35]. The method of obtaining CFS was not subject to MDT discussion, which represents a deviation from the original CFS validation cohort [3]. However, we suggest that our approach is comparable to use of the CFS in clinical practice [36]. The EQ-5D Summary Index for HRQOL is validated in UK populations, but the relationship to other quality of life measures is not clear. Finally, as with all observational data, we report associations rather than causation, and we must be cautious when applying these findings to the individual haemodialysis recipient in clinical practice.

To conclude, both somatic and cognitive components of depression are associated with frailty and poorer HRQOL in haemodialysis recipients, but they are not associated with mortality or hospitalisation on fully adjusted models including frailty. These data may suggest that there is an overlap between frailty and depression in their associations with negative patient outcomes. Further work is warranted to better understand and distinguish individual versus cumulative contributions from overlapping comorbidities towards adverse outcomes in prevalent haemodialysis patients.

## Figures and Tables

**Table 1:** Baseline demographics stratified by frailty status.

		Total cohort	Not frail	Frail
Frail^*∗*^		261	—	—
		53.8%	—	—

Median age		63	60	65
		53–74	50–72	55–76

Median CFS score		5	3	5
		3–6	3–4	5-6

Median PHQ-9 score		5	3	7
		2–10	1–7	3–12

Moderate depression^*∗∗*^		127	33	94
		26.5%	14.8%	36.6%

Median PHQ-9 somatic score		3	2	4
		1–6	1–4	2–6

Median PHQ-9 cognitive score		1	1	3
		0–5	0–3	0–6

Median MoCA		22	23	20
		17–25	20–26	16–23

Median albumin (g/L)		39	39	38
		35–41	36–42	34–41

Median BMI		26.8	26	27.9
		23.2–32.3	23.0–30.7	23.2–33.7

Median Charlson score^*∗∗∗*^		4	4	5
		3–6	2–5	4–7

Median HD vintage (months)		37	33	41
		17–76	13–66	19.9–81.5

Median Kt/V		1.59	1.58	1.61
		1.39–1.85	1.38–1.80	1.41–1.88

Median EQ summary index		0.779	1	0.62
		0.516–1.00	0.779–1.00	0.189–0.796

Health change	Better	94	52	42
		19.4%	23.2%	16.1%
	The Same	174	90	84
		35.9%	40.2%	32.2%
	Worse	217	82	135
		44.7%	36.6%	51.7%

IMD quintile	1	212	96	116
		43.7%	42.9%	44.4%
	2	87	43	44
		17.9%	19.2%	16.9%
	3	85	38	47
		17.5%	17.0%	18.0%
	4	38	20	18
		7.8%	8.9%	6.9%
	5	33	14	19
		6.8%	6.3%	7.3%
	Unknown	30	13	17
		6.2%	5.8%	6.5%

Ethnicity	White	281	13	144
		57.9%	61.2%	55.2%
	South Asian	115	44	71
		23.7%	19.6%	27.2%
	Black	76	35	41
		15.7%	15.6%	15.7%
	Other	13	8	5
		2.7%	3.6%	1.9%

Gender	Male	284	148	136
		58.6%	66.1%	52.1%

Comorbidities	Diabetes	138	43	95
		28.5%	19.2%	36.4%
	MI	98	34	64
		20.2%	15.2%	24.5%
	CVA/TIA	57	17	40
		11.8%	7.6%	15.3%
	Cancer	56	30	26
		11.6%	13.4%	10.0%
	Heart failure	52	19	33
		10.7%	8.5%	12.6%
	PVD	47	15	32
		9.7%	6.7%	12.3%

Primary renal disease	Diabetic	114	33	81
		23.5%	14.7%	31.3%
	Hypertensive	39	22	17
		8.0%	9.8%	6.5%
	Ischaemic	38	14	24
		7.8%	6.3%	9.2%
	IgA	37	20	17
		7.6%	8.9%	6.5%
	PKD	28	17	11
		5.8%	7.6%	4.2%
	FSGS	24	14	10
		5.0%	6.3%	3.8%
	Reflux	17	7	10
		3.5%	3.1%	3.8%
	Obstructive	16	10	6
		3.3%	4.5%	2.3%
	AAV	15	11	4
		3.1%	4.9%	1.5%
	Interstitial nephritis	10	6	4
		2.1%	2.7%	1.5%
	Myeloma	10	8	2
		2.1%	3.6%	0.8%
	Unknown	68	31	37
		14.0%	13.8%	14.2%

Smoking status	Current	68	38	30
		14.1%	17.0%	11.5%
	Ex	132	64	68
		27.3%	28.6%	26.2%
	Never	284	122	162
		58.7%	54.5%	62.3%

Dialysis access	Line	113	47	66
		23.3%	21.0%	25.3%

Transplant list status	Active	58	36	22
		12.0%	16.1%	8.4%
	Suspended	15	9	6
		3.1%	4.0%	2.3%
	Not listed	412	179	233
		85.0%	79.9%	89.3%

Employment status	Employed	69	61	8
		14.3%	27.2%	3.1%
	Unemployed	148	58	90
		30.6%	25.9%	34.6%
	Retired	267	105	162
		55.2%	46.9%	62.3%

Job role†	Unskilled manual	181	70	111
		39.3%	32.1%	45.7%
	Skilled manual	101	50	51
		21.9%	22.9%	21.0%
	Clerical	52	28	24
		11.3%	12.8%	9.9%
	Managerial	46	26	20
		10.0%	11.9%	8.2%
	Professional	81	44	37
		17.6%	20.2%	15.2%

Education level	High School	342	146	196
		70.7%	65.2%	75.4%
	College/Sixth Form	92	49	43
		19.0%	21.9%	16.5%
	University	50	29	21
		10.3%	13.0%	8.1%

Residence	Own home	462	218	244
		95.9%	97.8%	94.2%
	Warden-controlled	12	3	9
		2.5%	1.4%	3.5%
	Residential home	5	2	3
		1.0%	0.9%	1.2%
	Nursing home	3	0	3
		0.6%	0.0%	1.2%

All values *n* and percentages except where the median stated (median and interquartile range); ^*∗*^Frail = CFS ≥5; ^*∗∗*^PHQ-9 score ≥10; ^*∗∗∗*^CKD omitted; and †or previous occupation if unemployed/retired.

**Table 2:** Incidence rate ratios of hospital admissions associated with the PHQ-9 somatic component score. Fully adjusted model including CFS.

	HR	Lower 95% C.I.	Upper 95% C.I.	*P*
PHQ-9 somatic score	1.00	0.959	1.04	0.993
Age	**1.12**	**1.02**	**1.23**	**0.019**
CFS	**0.989**	**0.978**	**1.00**	**0.044**
Gender				
Male	REFERENCE			
Female	0.969	0.785	1.20	0.770
Ethnicity				
White	REFERENCE			
South Asian	0.826	0.622	1.10	0.188
Black	**0.687**	**0.504**	**0.937**	**0.018**
Other	0.848	0.436	1.65	0.628
BMI	0.998	0.983	1.01	0.757
IMD quintile				
1	REFERENCE			
2	0.887	0.664	1.18	0.415
3	0.829	0.610	1.13	0.231
4	0.808	0.542	1.21	0.298
5	0.771	0.495	1.20	0.250
Unknown	1.25	0.830	1.87	0.289
Charlson index	**1.09**	**1.03**	**1.17**	**0.006**
Previous admissions	**1.10**	**1.05**	**1.16**	**<0.001**
Medication number	1.02	0.989	1.05	0.208
Smoking status				
Current smoker	REFERENCE			
Ex-smoker	0.842	0.598	1.18	0.324
Never smoked	0.764	0.560	1.04	0.090
Albumin	0.998	0.994	1.00	0.155
Walking aid use				
No	REFERENCE			
Yes	**1.50**	**1.17**	**1.91**	**0.001**
HD vintage	1.000	0.998	1.00	0.968
Transplant listed				
No	REFERENCE			
Yes	0.903	0.633	1.29	0.575
Constant	**0.003**	**0.001**	**0.007**	**<0.001**

Incidence rate ratios obtained by negative binomial regression. Bold text indicates significance at the *P* < 0.05 level.

**Table 3:** Incidence rate ratios of hospital admissions associated with the PHQ-9 cognitive component score. Fully adjusted model including CFS.

	HR	Lower 95% C.I.	Upper 95% C.I.	*P*
PHQ-9 cognitive score	0.980	0.950	1.01	0.194
Age	**1.14**	**1.04**	**1.26**	**0.007**
CFS	**0.987**	**0.977**	**0.998**	**0.021**
Gender				
Male	REFERENCE			
Female	0.971	0.788	1.20	0.785
Ethnicity				
White	REFERENCE			
South Asian	0.802	0.604	1.06	0.126
Black	**0.670**	**0.490**	**0.914**	**0.012**
Other	0.822	0.423	1.60	0.564
BMI	0.997	0.983	1.01	0.701
IMD quintile				
1	REFERENCE			
2	0.871	0.652	1.16	0.349
3	0.823	0.605	1.12	0.212
4	0.802	0.538	1.20	0.279
5	0.756	0.485	1.18	0.215
Unknown	1.26	0.838	1.89	0.268
Charlson index	**1.09**	**1.02**	**1.16**	**0.007**
Previous admissions	**1.10**	**1.05**	**1.16**	**<0.001**
Medication number	1.02	0.992	1.05	0.146
Smoking status				
Current smoker	REFERENCE			
Ex-smoker	0.840	0.598	1.18	0.318
Never smoked	0.762	0.558	1.04	0.086
Albumin	0.998	0.994	1.00	0.158
Walking aid use				
No	REFERENCE			
Yes	**1.49**	**1.17**	**1.91**	**0.001**
HD vintage	1.00	0.998	1.00	0.944
Transplant listed				
No	REFERENCE			
Yes	0.896	0.628	1.28	0.546
Constant	**0.003**	**0.001**	**0.008**	**<0.001**

Incidence rate ratios obtained by negative binomial regression. Bold text indicates significance at the *P*<0.05 level.

**Table 4:** Fractional regression coefficients of somatic and cognitive component PHQ-9 scores upon EQ summary index.

PHQ-9 component	Fractional regression model	Coefficient	Lower 95% C.I.	Upper 95% C.I.	*P*
Somatic	Univariable	−0.110	−0.146	−0.073	<0.001
	1	−0.093	−0.130	−0.056	<0.001
	2	−0.096	−0.135	−0.058	<0.001
	3	−0.102	−0.139	−0.064	<0.001
	4	−0.062	−0.104	−0.021	0.003

Cognitive	Univariable	−0.077	−0.102	−0.052	<0.001
	1	−0.071	−0.098	−0.043	<0.001
	2	−0.078	−0.106	−0.049	<0.001
	3	−0.084	−0.112	−0.057	<0.001
	4	−0.052	−0.081	−0.024	<0.001

Obtained by fractional regression. Coefficient:  change in the EQ fractional summary index score for each 1-point rise in PHQ-9 somatic or cognitive component scores. Univariable and adjusted models are shown. Model 1 included age, gender, ethnicity, education level, social support, IMD quintile, and employment status. Model 2 added to these haemodialysis vintage, Charlson comorbidity index (CKD omitted), haemoglobin, Kt/V, and current use of antidepressant medication. Model 3 added use of walking aids, slow walking (or inability to walk), and physical activity index. Model 4 added EQ self-rated health today (continuous score from 0 to 100) and self-rated health change.

## Data Availability

Data underlying this manuscript will be made available from the corresponding author upon reasonable request.

## Additional Points

We performed a prospective cohort study investigating the relationship between symptom subsets of the PHQ-9 score with the Clinical Frailty Scale in prevalent haemodialysis recipients. Both somatic and cognitive component scores of PHQ-9 were associated with frailty and poorer HRQOL but were not associated with mortality or hospitalisation when adjusted for frailty.

## Supplementary Materials

Supplementary Materials Supplementary Table 1: multiple linear regression of the CFS score associated with the PHQ-9 somatic score. Fully adjusted model 4. Supplementary Table 2: multiple linear regression of the CFS score associated with the PHQ-9 cognitive score. Fully adjusted model 4. Supplementary Table 3: fully adjusted Cox regression model of mortality associated with the PHQ-9 somatic component score. CFS omitted. Supplementary Table 4: fully adjusted Cox regression of mortality associated with the PHQ-9 somatic component score. CFS included. Supplementary Table 5: fully adjusted Cox regression of mortality associated with the PHQ-9 cognitive component score. CFS omitted. Supplementary Table 6: fully adjusted Cox regression model of mortality associated with the PHQ-9 cognitive component score. CFS included. Supplementary Table 7: incidence rate ratios of hospital admissions associated with the PHQ-9 somatic component score. Adjusted model with CFS omitted. Supplementary Table 8: incidence rate ratios of hospital admissions associated with the PHQ-9 cognitive component score. Adjusted model with CFS omitted. Supplementary Table 9: fractional regression of association of PHQ-9 somatic component scores with EQ Summary Index: fully adjusted model 4. Supplementary Table 10: fractional regression of association of PHQ-9 cognitive component scores with EQ Summary Index: fully adjusted model 4.
