# Supplementary material for: Somatic Symptoms of Depression Lose Association with Mortality upon Adjustment for Frailty: Analysis from the Fitness Haemodialysis Cohort
Source: Int J Nephrol. 2023 Jun 21;2023:4518843. doi: 10.1155/2023/4518843 (PMC10307017; doi:10.1155/2023/4518843)
Supplement: Supplementary Materials — Supplementary Table 1: multiple linear regression of the CFS score associated with the PHQ-9 somatic score. Fully adjusted model 4. Supplementary Table 2: multiple linear regression of the CFS score associated with the PHQ-9 cognitive score. Fully adjusted model 4. Supplementary Table 3: fully adjusted Cox regression model of mortality associated with the PHQ-9 somatic component score. CFS omitted. Supplementary Table 4: fully adjusted Cox regression of mortality associated with the PHQ-9 somatic component score. CFS included. Supplementary Table 5: fully adjusted Cox regression of mortality associated with the PHQ-9 cognitive component score. CFS omitted. Supplementary Table 6: fully adjusted Cox regression model of mortality associated with the PHQ-9 cognitive component score. CFS included. Supplementary Table 7: incidence rate ratios of hospital admissions associated with the PHQ-9 somatic component score. Adjusted model with CFS omitted. Supplementary Table 8: incidence rate ratios of hospital admissions associated with the PHQ-9 cognitive component score. Adjusted model with CFS omitted. Supplementary Table 9: fractional regression of association of PHQ-9 somatic component scores with EQ Summary Index: fully adjusted model 4. Supplementary Table 10: fractional regression of association of PHQ-9 cognitive component scores with EQ Summary Index: fully adjusted model 4. [file 4518843.f1.docx]

Supplementary Table 1: Multiple linear regression of CFS score associated with PHQ-9 Somatic Score. Fully adjusted model 4.

|  | Coef. | Lower 95% C.I. | Upper 95% C.I. | P |
| --- | --- | --- | --- | --- |
| PHQ-9 somatic score | **0.067** | **0.029** | **0.104** | **<0.001** |
| Age | **-0.011** | **-0.020** | **-0.002** | **0.019** |
| Gender |  |  |  |  |
| *Male* | REFERENCE | | | |
| *Female* | **0.224** | **0.037** | **0.412** | **0.019** |
| Ethnicity |  |  |  |  |
| *White* | REFERENCE | | | |
| *South Asian* | 0.181 | -0.072 | 0.434 | 0.160 |
| *Black* | -0.048 | -0.314 | 0.218 | 0.724 |
| *Other* | 0.274 | -0.320 | 0.868 | 0.365 |
| Education Level |  |  |  |  |
| *High School* | REFERENCE | | | |
| *College/6th form* | -0.009 | -0.242 | 0.224 | 0.937 |
| *University* | -0.211 | -0.529 | 0.106 | 0.191 |
| IMD Quintile |  |  |  |  |
| *1* | REFERENCE | | | |
| *2* | 0.109 | -0.144 | 0.361 | 0.398 |
| *3* | -0.002 | -0.258 | 0.254 | 0.989 |
| *4* | **-0.354** | **-0.703** | **-0.005** | **0.047** |
| *5* | -0.003 | -0.376 | 0.370 | 0.987 |
| *Unknown* | 0.212 | -0.182 | 0.606 | 0.291 |
| Social Support |  |  |  |  |
| *Yes* | REFERENCE | | | |
| *No* | **-0.687** | **-1.06** | **-0.316** | **<0.001** |
| Charlson Index* | **0.094** | **0.040** | **0.148** | **0.001** |
| Cognitive Impairment |  |  |  |  |
| *No* | REFERENCE | | | |
| *Yes* | 0.011 | -0.220 | 0.243 | 0.923 |
| Smoking Status |  |  |  |  |
| *Current Smoker* | REFERENCE | | | |
| *Ex-Smoker* | **0.294** | **0.004** | **0.585** | **0.047** |
| *Never Smoked* | 0.197 | -0.076 | 0.469 | 0.157 |
| Health Change |  |  |  |  |
| *Better/The Same* | REFERENCE | | | |
| *Worse* | 0.112 | -0.080 | 0.305 | 0.252 |
| EQ VAS Score | **-0.008** | **-0.013** | **-0.004** | **<0.001** |
| Walking Aid Use |  |  |  |  |
| *No* | REFERENCE | | | |
| *Yes* | **0.517** | **0.291** | **0.743** | **<0.001** |
| Physical Activity Index |  |  |  |  |
| *Inactive* | REFERENCE | | | |
| *Moderately Inactive* | **-0.408** | **-0.760** | **-0.056** | **0.023** |
| *Moderately Active* | **-1.07** | **-1.49** | **-0.648** | **<0.001** |
| *Active* | **-1.92** | **-2.36** | **-1.48** | **<0.001** |
| Walking Speed |  |  |  |  |
| *Not Slow* | REFERENCE | | | |
| *Slow* | **0.721** | **0.491** | **0.951** | **<0.001** |
| Grip Strength |  |  |  |  |
| *Not Weak* | REFERENCE | | | |
| *Weak* | 0.075 | -0.128 | 0.279 | 0.466 |
| Constant | **4.22** | **3.59** | **4.85** | **<0.001** |

Supplementary Table 2: Multiple linear regression of CFS score associated with PHQ-9 Cognitive Score. Fully adjusted model 4.

|  | Coef. | Lower 95% C.I. | Upper 95% C.I. | P |
| --- | --- | --- | --- | --- |
| PHQ-9 Cognitive score | **0.062** | **0.034** | **0.089** | **<0.001** |
| Age | -0.008 | -0.017 | 0.001 | 0.072 |
| Gender |  |  |  |  |
| *Male* | REFERENCE | | | |
| *Female* | **0.249** | **0.064** | **0.433** | **0.008** |
| Ethnicity |  |  |  |  |
| *White* | REFERENCE | | | |
| *South Asian* | 0.208 | -0.044 | 0.459 | 0.105 |
| *Black* | 0.018 | -0.247 | 0.284 | 0.891 |
| *Other* | 0.259 | -0.330 | 0.848 | 0.388 |
| Education Level |  |  |  |  |
| *High School* | REFERENCE | | | |
| *College/6th form* | -0.002 | -0.233 | 0.230 | 0.989 |
| *University* | -0.172 | -0.488 | 0.144 | 0.286 |
| IMD Quintile |  |  |  |  |
| *1* | REFERENCE | | | |
| *2* | 0.094 | -0.156 | 0.344 | 0.461 |
| *3* | 0.024 | -0.230 | 0.278 | 0.854 |
| *4* | -0.273 | -0.621 | 0.075 | 0.124 |
| *5* | 0.004 | -0.366 | 0.374 | 0.982 |
| *Unknown* | 0.221 | -0.170 | 0.612 | 0.267 |
| Social Support |  |  |  |  |
| *Yes* | REFERENCE | | | |
| *No* | **-0.683** | **-1.05** | **-0.316** | **<0.001** |
| Charlson Index* | **0.095** | **0.041** | **0.148** | **0.001** |
| Cognitive Impairment |  |  |  |  |
| *No* | REFERENCE | | | |
| *Yes* | -0.003 | -0.233 | 0.226 | 0.977 |
| Smoking Status |  |  |  |  |
| *Current Smoker* | REFERENCE | | | |
| *Ex-Smoker* | 0.279 | -0.009 | 0.568 | 0.058 |
| *Never Smoked* | 0.151 | -0.120 | 0.423 | 0.274 |
| Health Change |  |  |  |  |
| *Better/The Same* | REFERENCE | | | |
| *Worse* | 0.114 | -0.076 | 0.303 | 0.240 |
| EQ VAS Score | **-0.007** | **-0.011** | **-0.002** | **0.004** |
| Walking Aid Use |  |  |  |  |
| *No* | REFERENCE | | | |
| *Yes* | **0.565** | **0.341** | **0.790** | **<0.001** |
| Physical Activity Index |  |  |  |  |
| *Inactive* | REFERENCE | | | |
| *Moderately Inactive* | **-0.391** | **-0.740** | **-0.042** | **0.028** |
| *Moderately Active* | **-1.03** | **-1.45** | **-0.610** | **<0.001** |
| *Active* | **-1.88** | **-2.32** | **-1.44** | **<0.001** |
| Walking Speed |  |  |  |  |
| *Not Slow* | REFERENCE | | | |
| *Slow* | **0.690** | **0.461** | **0.918** | **<0.001** |
| Grip Strength |  |  |  |  |
| *Not Weak* | REFERENCE | | | |
| *Weak* | 0.100 | -0.101 | 0.300 | 0.330 |
| Constant | **4.01** | **3.36** | **4.65** | **<0.001** |

Supplementary Table 3: Fully adjusted Cox regression model of mortality associated with PHQ-9 Somatic Component Score. CFS omitted.

|  | HR | Lower 95% C.I. | Upper 95% C.I. | P |
| --- | --- | --- | --- | --- |
| PHQ-9 Somatic Score | **1.08** | **1.00** | **1.17** | **0.038** |
| Age | 0.997 | 0.975 | 1.018 | 0.755 |
| Gender |  |  |  |  |
| *Male* | REFERENCE | | | |
| *Female* | 0.894 | 0.595 | 1.34 | 0.587 |
| Ethnicity |  |  |  |  |
| *White* | REFERENCE | | | |
| *South Asian* | 0.586 | 0.325 | 1.06 | 0.076 |
| *Black* | 0.948 | 0.543 | 1.66 | 0.852 |
| *Other* | 0.553 | 0.074 | 4.11 | 0.563 |
| BMI | 0.995 | 0.969 | 1.02 | 0.739 |
| IMD Quintile |  |  |  |  |
| *1* | REFERENCE | | | |
| *2* | 1.10 | 0.635 | 1.89 | 0.743 |
| *3* | 1.26 | 0.730 | 2.16 | 0.411 |
| *4* | 0.750 | 0.329 | 1.71 | 0.494 |
| *5* | 0.834 | 0.362 | 1.92 | 0.669 |
| *Unknown* | 0.945 | 0.369 | 2.42 | 0.907 |
| Charlson Index | **1.18** | **1.06** | **1.32** | **0.002** |
| Previous Admissions | 1.07 | 0.981 | 1.17 | 0.128 |
| Medication Number | 1.01 | 0.955 | 1.07 | 0.706 |
| Smoking Status |  |  |  |  |
| *Current Smoker* | REFERENCE | | | |
| *Ex-Smoker* | 1.28 | 0.686 | 2.38 | 0.439 |
| *Never Smoked* | 0.851 | 0.467 | 1.55 | 0.599 |
| Albumin | **0.944** | **0.907** | **0.983** | **0.006** |
| Walking Aid Use |  |  |  |  |
| *No* | REFERENCE | | | |
| *Yes* | **1.73** | **1.07** | **2.78** | **0.024** |
| HD vintage | 1.00 | 0.998 | 1.01 | 0.324 |
| Transplant Listed |  |  |  |  |
| *No* | REFERENCE | | | |
| *Yes* | 0.150 | 0.020 | 1.11 | 0.063 |

Supplementary Table 4: Fully adjusted Cox regression of mortality associated with PHQ-9 Somatic Component Score. CFS included.

|  | **HR** | **Lower 95% C.I.** | **Upper 95% C.I.** | **P** |
| --- | --- | --- | --- | --- |
| PHQ-9 Somatic Score | 1.06 | 0.977 | 1.14 | 0.173 |
| Age | 0.998 | 0.977 | 1.02 | 0.844 |
| CFS | **1.26** | **1.05** | **1.53** | **0.015** |
| Gender |  |  |  |  |
| *Male* | REFERENCE | | | |
| *Female* | 0.838 | 0.557 | 1.26 | 0.397 |
| Ethnicity |  |  |  |  |
| *White* | REFERENCE | | | |
| *South Asian* | 0.560 | 0.311 | 1.01 | 0.054 |
| *Black* | 0.968 | 0.554 | 1.69 | 0.908 |
| *Other* | 0.516 | 0.069 | 3.86 | 0.519 |
| BMI | 0.993 | 0.966 | 1.02 | 0.600 |
| IMD Quintile |  |  |  |  |
| *1* | REFERENCE | | | |
| *2* | 1.11 | 0.645 | 1.92 | 0.698 |
| *3* | 1.29 | 0.753 | 2.22 | 0.352 |
| *4* | 0.811 | 0.355 | 1.85 | 0.619 |
| *5* | 0.868 | 0.376 | 2.01 | 0.741 |
| *Unknown* | 0.974 | 0.379 | 2.50 | 0.955 |
| Charlson Index | **1.16** | **1.04** | **1.29** | **0.007** |
| Previous Admissions | 1.07 | 0.980 | 1.17 | 0.127 |
| Medication Number | 1.00 | 0.944 | 1.06 | 0.977 |
| Smoking Status |  |  |  |  |
| *Current Smoker* | REFERENCE | | | |
| *Ex-Smoker* | 1.25 | 0.673 | 2.34 | 0.475 |
| *Never Smoked* | 0.809 | 0.443 | 1.48 | 0.491 |
| Albumin | **0.948** | **0.910** | **0.987** | **0.009** |
| Walking Aid Use |  |  |  |  |
| *No* | REFERENCE | | | |
| *Yes* | 1.37 | 0.824 | 2.29 | 0.223 |
| HD vintage | 1.00 | 0.998 | 1.00 | 0.387 |
| Transplant Listed |  |  |  |  |
| *No* | REFERENCE | | | |
| *Yes* | 0.158 | 0.021 | 1.16 | 0.070 |

Supplementary Table 5: Fully adjusted Cox regression of mortality associated with PHQ-9 Cognitive Component Score. CFS omitted.

|  | HR | Lower 95% C.I. | Upper 95% C.I. | P |
| --- | --- | --- | --- | --- |
| PHQ-9 Cognitive Score | 0.996 | 0.941 | 1.05 | 0.877 |
| Age | 0.993 | 0.972 | 1.01 | 0.498 |
| Gender |  |  |  |  |
| *Male* | REFERENCE | | | |
| *Female* | 0.952 | 0.637 | 1.42 | 0.812 |
| Ethnicity |  |  |  |  |
| *White* | REFERENCE | | | |
| *South Asian* | 0.559 | 0.308 | 1.01 | 0.056 |
| *Black* | 0.926 | 0.528 | 1.62 | 0.788 |
| *Other* | 0.553 | 0.074 | 4.13 | 0.564 |
| BMI | 0.996 | 0.969 | 1.02 | 0.762 |
| IMD Quintile |  |  |  |  |
| *1* | REFERENCE | | | |
| *2* | 1.02 | 0.593 | 1.76 | 0.936 |
| *3* | 1.21 | 0.698 | 2.09 | 0.499 |
| *4* | 0.730 | 0.319 | 1.67 | 0.457 |
| *5* | 0.760 | 0.331 | 1.74 | 0.517 |
| *Unknown* | 0.933 | 0.363 | 2.40 | 0.885 |
| Charlson Index | **1.18** | **1.06** | **1.32** | **0.003** |
| Previous Admissions | 1.07 | 0.978 | 1.16 | 0.147 |
| Medication Number | 1.03 | 0.970 | 1.09 | 0.356 |
| Smoking Status |  |  |  |  |
| *Current Smoker* | REFERENCE | | | |
| *Ex-Smoker* | 1.32 | 0.712 | 2.45 | 0.376 |
| *Never Smoked* | 0.854 | 0.467 | 1.56 | 0.608 |
| Albumin | **0.947** | **0.909** | **0.986** | **0.008** |
| Walking Aid Use |  |  |  |  |
| *No* | REFERENCE | | | |
| *Yes* | **1.81** | **1.13** | **2.91** | **0.014** |
| HD vintage | 1.00 | 0.998 | 1.01 | 0.339 |
| Transplant Listed |  |  |  |  |
| *No* | REFERENCE | | | |
| *Yes* | 0.145 | 0.020 | 1.07 | 0.059 |

Supplementary Table 6: Fully adjusted Cox regression model of mortality associated with PHQ-9 Cognitive Component Score. CFS included.

|  | **HR** | **Lower 95% C.I.** | **Upper 95% C.I.** | **P** |
| --- | --- | --- | --- | --- |
| PHQ-9 Cognitive Score | 0.967 | 0.910 | 1.03 | 0.270 |
| Age | 0.993 | 0.972 | 1.01 | 0.503 |
| CFS | **1.35** | **1.12** | **1.64** | **0.002** |
| Gender |  |  |  |  |
| *Male* | REFERENCE | | | |
| *Female* | 0.871 | 0.582 | 1.30 | 0.503 |
| Ethnicity |  |  |  |  |
| *White* | REFERENCE | | | |
| *South Asian* | **0.517** | **0.286** | **0.935** | **0.029** |
| *Black* | 0.915 | 0.521 | 1.61 | 0.759 |
| *Other* | 0.492 | 0.066 | 3.69 | 0.490 |
| BMI | 0.992 | 0.965 | 1.02 | 0.555 |
| IMD Quintile |  |  |  |  |
| *1* | REFERENCE | | | |
| *2* | 1.02 | 0.589 | 1.76 | 0.948 |
| *3* | 1.23 | 0.713 | 2.12 | 0.456 |
| *4* | 0.766 | 0.335 | 1.75 | 0.527 |
| *5* | 0.792 | 0.344 | 1.82 | 0.584 |
| *Unknown* | 0.987 | 0.384 | 2.54 | 0.979 |
| Charlson Index | **1.16** | **1.04** | **1.29** | **0.010** |
| Previous Admissions | 1.07 | 0.978 | 1.17 | 0.140 |
| Medication Number | 1.01 | 0.954 | 1.07 | 0.708 |
| Smoking Status |  |  |  |  |
| *Current Smoker* | REFERENCE | | | |
| *Ex-Smoker* | 1.30 | 0.703 | 2.42 | 0.400 |
| *Never Smoked* | 0.825 | 0.450 | 1.51 | 0.533 |
| Albumin | **0.951** | **0.913** | **0.989** | **0.013** |
| Walking Aid Use |  |  |  |  |
| *No* | REFERENCE | | | |
| *Yes* | 1.36 | 0.817 | 2.26 | 0.238 |
| HD vintage | 1.00 | 0.998 | 1.00 | 0.482 |
| Transplant Listed |  |  |  |  |
| *No* | REFERENCE | | | |
| *Yes* | 0.154 | 0.021 | 1.14 | 0.067 |

Supplementary Table 7: Incidence Rate Ratios of hospital admissions associated with PHQ-9 somatic component score. Adjusted model with CFS omitted.

|  | IRR | Lower 95% C.I. | Upper 95% C.I. | P |
| --- | --- | --- | --- | --- |
| PHQ-9 Somatic Score | 1.01 | 0.97 | 1.05 | 0.559 |
| Age | **0.99** | **0.98** | **1.00** | **0.043** |
| Gender |  |  |  |  |
| *Male* | REFERENCE | | | |
| *Female* | 0.997 | 0.808 | 1.23 | 0.978 |
| Ethnicity |  |  |  |  |
| *White* | REFERENCE | | | |
| *South Asian* | 0.857 | 0.645 | 1.14 | 0.286 |
| *Black* | **0.679** | **0.497** | **0.927** | **0.015** |
| *Other* | 0.879 | 0.451 | 1.71 | 0.704 |
| BMI | 0.998 | 0.983 | 1.01 | 0.786 |
| IMD Quintile |  |  |  |  |
| *1* | REFERENCE | | | |
| *2* | 0.882 | 0.660 | 1.18 | 0.400 |
| *3* | 0.820 | 0.602 | 1.12 | 0.207 |
| *4* | 0.777 | 0.520 | 1.16 | 0.219 |
| *5* | 0.735 | 0.471 | 1.15 | 0.175 |
| *Unknown* | 1.23 | 0.816 | 1.85 | 0.324 |
| Charlson Index | **1.10** | **1.04** | **1.18** | **0.002** |
| Previous Admissions | **1.10** | **1.05** | **1.16** | **<0.001** |
| Medication Number | 1.02 | 0.994 | 1.06 | 0.117 |
| Smoking Status |  |  |  |  |
| *Current Smoker* | REFERENCE | | | |
| *Ex-Smoker* | 0.855 | 0.606 | 1.21 | 0.374 |
| *Never Smoked* | 0.785 | 0.574 | 1.07 | 0.129 |
| Albumin | 0.997 | 0.994 | 1.00 | 0.118 |
| Walking Aid Use |  |  |  |  |
| *No* | REFERENCE | | | |
| *Yes* | **1.70** | **1.35** | **2.12** | **<0.001** |
| HD vintage | 1.00 | 0.998 | 1.00 | 0.962 |
| Transplant Listed |  |  |  |  |
| *No* | REFERENCE | | | |
| *Yes* | 0.893 | 0.624 | 1.28 | 0.537 |
| Constant | **0.004** | **0.002** | **0.009** | **<0.001** |

Supplementary Table 8: Incidence Rate Ratios of hospital admissions associated with PHQ-9 cognitive component score. Adjusted model with CFS omitted.

|  | IRR | Lower 95% C.I. | Upper 95% C.I. | P |
| --- | --- | --- | --- | --- |
| PHQ-9 Cognitive Score | 0.994 | 0.965 | 1.02 | 0.707 |
| Age | **0.988** | **0.977** | **0.999** | **0.027** |
| Gender |  |  |  |  |
| *Male* | REFERENCE | | | |
| *Female* | 1.01 | 0.817 | 1.24 | 0.948 |
| Ethnicity |  |  |  |  |
| *White* | REFERENCE | | | |
| *South Asian* | 0.841 | 0.634 | 1.12 | 0.231 |
| *Black* | 0.672 | 0.491 | 0.920 | 0.013 |
| *Other* | 0.862 | 0.442 | 1.68 | 0.662 |
| BMI | 0.998 | 0.983 | 1.01 | 0.778 |
| IMD Quintile |  |  |  |  |
| *1* | REFERENCE | | | |
| *2* | 0.870 | 0.650 | 1.16 | 0.351 |
| *3* | 0.814 | 0.598 | 1.11 | 0.193 |
| *4* | 0.778 | 0.521 | 1.16 | 0.221 |
| *5* | 0.721 | 0.462 | 1.13 | 0.151 |
| *Unknown* | 1.23 | 0.816 | 1.85 | 0.322 |
| Charlson Index | **1.10** | **1.04** | **1.18** | **0.002** |
| Previous Admissions | **1.10** | **1.05** | **1.15** | **<0.001** |
| Medication Number | 1.03 | 0.998 | 1.06 | 0.066 |
| Smoking Status |  |  |  |  |
| *Current Smoker* | REFERENCE | | | |
| *Ex-Smoker* | 0.854 | 0.605 | 1.21 | 0.369 |
| *Never Smoked* | 0.784 | 0.573 | 1.07 | 0.127 |
| Albumin | 0.997 | 0.994 | 1.00 | 0.126 |
| Walking Aid Use |  |  |  |  |
| *No* | REFERENCE | | | |
| *Yes* | **1.72** | **1.37** | **2.16** | **<0.001** |
| HD vintage | 1.000 | 0.998 | 1.00 | 0.927 |
| Transplant Listed |  |  |  |  |
| *No* | REFERENCE | | | |
| *Yes* | 0.884 | 0.618 | 1.26 | 0.499 |
| Constant | **0.005** | **0.002** | **0.010** | **<0.001** |

Supplementary Table 9: Fractional Regression of association of PHQ-9 Somatic Component scores with EQ Summary Index: Fully adjusted Model 4

|  | Coefficient | Lower 95% C.I. | Upper 95% C.I. | P |
| --- | --- | --- | --- | --- |
| PHQ-9 Somatic Score | **-0.062** | **-0.104** | **-0.021** | **0.003** |
| CFS | **-0.419** | **-0.524** | **-0.313** | **<0.001** |
| Age | 0.003 | -0.010 | 0.017 | 0.631 |
| Gender |  |  |  |  |
| *Male* | REFERENCE | | | |
| *Female* | **-0.220** | **-0.433** | **-0.007** | **0.043** |
| Ethnicity |  |  |  |  |
| *White* | REFERENCE | | | |
| *South Asian* | **0.305** | **0.009** | **0.601** | **0.043** |
| *Black* | 0.173 | -0.149 | 0.496 | 0.292 |
| *Other* | -0.498 | -1.219 | 0.224 | 0.176 |
| Education Level |  |  |  |  |
| *High School* | REFERENCE | | | |
| *College/6th form* | -0.244 | -0.537 | 0.048 | 0.102 |
| *University* | **-0.339** | **-0.647** | **-0.031** | **0.031** |
| Social Support? |  |  |  |  |
| *Yes* | REFERENCE | | | |
| *No* | **-0.507** | **-0.844** | **-0.169** | **0.003** |
| IMD Quintile |  |  |  |  |
| *1* | REFERENCE | | | |
| *2* | -0.210 | -0.506 | 0.087 | 0.166 |
| *3* | 0.043 | -0.258 | 0.344 | 0.779 |
| *4* | 0.188 | -0.213 | 0.590 | 0.358 |
| *5* | 0.283 | -0.196 | 0.762 | 0.246 |
| *Unknown* | 0.184 | -0.322 | 0.690 | 0.475 |
| Employment Status |  |  |  |  |
| *Employed* | REFERENCE | | | |
| *Unemployed* | -0.034 | -0.711 | 0.642 | 0.921 |
| *Retired* | 0.133 | -0.469 | 0.735 | 0.665 |
| HD Vintage (months) | **-0.003** | **-0.005** | **-0.001** | **0.012** |
| Charlson Comorbidity Index* | -0.011 | -0.068 | 0.047 | 0.717 |
| Haemoglobin (g/L) | 0.003 | -0.007 | 0.012 | 0.585 |
| Kt/V | 0.155 | -0.147 | 0.457 | 0.315 |
| Antidepressant Use? |  |  |  |  |
| *No* | REFERENCE | | | |
| *Yes* | -0.154 | -0.501 | 0.193 | 0.384 |
| Walking Aid Use? |  |  |  |  |
| *No* | REFERENCE | | | |
| *Yes* | -0.170 | -0.421 | 0.082 | 0.185 |
| Slow walk speed? |  |  |  |  |
| *No* | REFERENCE | | | |
| *Yes* | **-0.464** | **-0.730** | **-0.198** | **0.001** |
| Physical Activity Index |  |  |  |  |
| *Inactive* | REFERENCE | | | |
| *Moderately Inactive* | -0.076 | -0.751 | 0.598 | 0.825 |
| *Moderately Active* | **-0.793** | **-1.397** | **-0.188** | **0.010** |
| *Active* | -0.084 | -0.959 | 0.792 | 0.852 |
| Self-reported health today (/100)** | **0.014** | **0.009** | **0.019** | **<0.001** |
| Self-reported health change*** |  |  |  |  |
| *Better* | REFERENCE | | | |
| *The Same* | 0.134 | -0.166 | 0.433 | 0.382 |
| *Worse* | -0.100 | -0.385 | 0.185 | 0.491 |
| Cognitive Impairment |  |  |  |  |
| *No* | REFERENCE | | | |
| *Yes* | **-0.305** | **-0.569** | **-0.040** | **0.024** |
| Constant | **3.080** | **1.275** | **4.885** | **0.001** |

Supplementary Table 10: Fractional Regression of association of PHQ-9 Cognitive Component scores with EQ Summary Index: Fully adjusted Model 4

|  | Coefficient | Lower 95% C.I. | Upper 95% C.I. | P |
| --- | --- | --- | --- | --- |
| PHQ-9 Cognitive Score | **-0.052** | **-0.081** | **-0.024** | **<0.001** |
| CFS | **-0.410** | **-0.517** | **-0.303** | **<0.001** |
| Age | 0.003 | -0.011 | 0.016 | 0.716 |
| Gender |  |  |  |  |
| *Male* | REFERENCE | | | |
| *Female* | **-0.254** | **-0.461** | **-0.046** | **0.017** |
| Ethnicity |  |  |  |  |
| *White* | REFERENCE | | | |
| *South Asian* | 0.270 | -0.031 | 0.570 | 0.079 |
| *Black* | 0.146 | -0.176 | 0.468 | 0.373 |
| *Other* | -0.476 | -1.238 | 0.286 | 0.221 |
| Education Level |  |  |  |  |
| *High School* | REFERENCE | | | |
| *College/6th form* | -0.272 | -0.559 | 0.016 | 0.064 |
| *University* | **-0.378** | **-0.694** | **-0.062** | **0.019** |
| Social Support? |  |  |  |  |
| *Yes* | REFERENCE | | | |
| *No* | **-0.511** | **-0.836** | **-0.185** | **0.002** |
| IMD Quintile |  |  |  |  |
| *1* | REFERENCE | | | |
| *2* | -0.186 | -0.482 | 0.110 | 0.219 |
| *3* | 0.021 | -0.270 | 0.312 | 0.889 |
| *4* | 0.125 | -0.279 | 0.530 | 0.544 |
| *5* | 0.291 | -0.179 | 0.761 | 0.225 |
| *Unknown* | 0.156 | -0.346 | 0.658 | 0.543 |
| Employment Status |  |  |  |  |
| *Employed* | REFERENCE | | | |
| *Unemployed* | -0.042 | -0.737 | 0.654 | 0.906 |
| *Retired* | 0.056 | -0.568 | 0.680 | 0.861 |
| HD Vintage (months) | **-0.003** | **-0.005** | **-0.001** | **0.008** |
| Charlson Comorbidity Index* | -0.012 | -0.072 | 0.047 | 0.684 |
| Haemoglobin (g/L) | 0.005 | -0.004 | 0.014 | 0.317 |
| Kt/V | 0.250 | -0.049 | 0.549 | 0.101 |
| Antidepressant Use? |  |  |  |  |
| *No* | REFERENCE | | | |
| *Yes* | -0.132 | -0.453 | 0.190 | 0.422 |
| Walking Aid Use? |  |  |  |  |
| *No* | REFERENCE | | | |
| *Yes* | -0.216 | -0.465 | 0.032 | 0.088 |
| Slow walk speed? |  |  |  |  |
| *No* | REFERENCE | | | |
| *Yes* | **-0.444** | **-0.705** | **-0.184** | **0.001** |
| Physical Activity Index |  |  |  |  |
| *Inactive* | REFERENCE | | | |
| *Moderately Inactive* | -0.076 | -0.787 | 0.635 | 0.834 |
| *Moderately Active* | **-0.805** | **-1.415** | **-0.194** | **0.010** |
| *Active* | -0.093 | -0.978 | 0.791 | 0.836 |
| Self-reported health today (/100)** | **0.013** | **0.007** | **0.018** | **<0.001** |
| Self-reported health change*** |  |  |  |  |
| *Better* | REFERENCE | | | |
| *The Same* | 0.096 | -0.206 | 0.398 | 0.533 |
| *Worse* | -0.134 | -0.419 | 0.152 | 0.359 |
| Cognitive Impairment |  |  |  |  |
| *No* | REFERENCE | | | |
| *Yes* | **-0.302** | **-0.567** | **-0.038** | **0.025** |
| Constant | **2.852** | **1.073** | **4.631** | **0.002** |
